# Characterization of G6PD Genotypes and Phenotypes on the Northwestern Thailand-Myanmar Border

**DOI:** 10.1371/journal.pone.0116063

**Published:** 2014-12-23

**Authors:** Germana Bancone, Cindy S. Chu, Raweewan Somsakchaicharoen, Nongnud Chowwiwat, Daniel M. Parker, Prakaykaew Charunwatthana, Nicholas J. White, François H. Nosten

**Affiliations:** 1 Shoklo Malaria Research Unit, Mahidol-Oxford Tropical Medicine Research Unit, Faculty of Tropical Medicine, Mahidol University, Mae Sot, Thailand; 2 Mahidol-Oxford Tropical Medicine Research Unit, Faculty of Tropical Medicine, Mahidol University, Bangkok, Thailand; 3 Centre for Tropical Medicine, Nuffield Department of Medicine, University of Oxford, Oxford, United Kingdom; Agency for Science, Technology and Research - Singapore Immunology Network, Singapore

## Abstract

Mutations in the glucose-6-phosphate dehydrogenase (G6PD) gene result in red blood cells with increased susceptibility to oxidative damage. Significant haemolysis can be caused by primaquine and other 8-aminoquinoline antimalarials used for the radical treatment of *Plasmodium vivax* malaria. The distribution and phenotypes of mutations causing G6PD deficiency in the male population of migrants and refugees in a malaria endemic region on the Thailand-Myanmar border were characterized. Blood samples for G6PD fluorescent spot test (FST), G6PD genotyping, and malaria testing were taken from 504 unrelated males of Karen and Burman ethnicities presenting to the outpatient clinics. The overall frequency of G6PD deficiency by the FST was 13.7%. Among the deficient subjects, almost 90% had the Mahidol variant (487G>A) genotype. The remaining subjects had Chinese-4 (392G>T), Viangchan (871G>A), Açores (595A>G), Seattle (844G>C) and Mediterranean (563C>T) variants. Quantification of G6PD activity was performed using a modification of the standard spectrophotometric assay on a subset of 24 samples with Mahidol, Viangchan, Seattle and Chinese-4 mutations; all samples showed a residual enzymatic activity below 10% of normal and were diagnosed correctly by the FST. Further studies are needed to characterise the haemolytic risk of using 8-aminoquinolines in patients with these genotypes.

## Introduction

Glucose-6-phosphate dehydrogenase (G6PD) deficiency is the most common genetic disorder in humans. It is prevalent throughout Africa, Asia, Southeast Asia and parts of South America, where malaria is endemic [1]. Different mutations in the gene encoding for G6PD cause varying degrees of protein instability that renders the mature red blood cells more susceptible to oxidative damage. Antimalarial drugs such as the 8-aminoquinolines (primaquine, tafenoquine) cause dose-dependent damage to G6PD deficient red blood cells resulting in intravascular haemolysis and removal of damaged erythrocytes by the spleen. The 8-aminoquinoline drugs are essential for the radical cure of *Plasmodium vivax* infections and for blocking the transmission of *Plasmodium falciparum*. The degree of haemolysis associated with the use of these drugs has been analyzed in few genetic variants and very limited data exist in most populations on either the hemolytic risk associated with a specific drug regimen in subjects with a specific G6PD variant or on the interaction with other genetic and clinical factors. Mutations in the G6PD gene located on the X chromosome usually result in reduced enzyme stability (and thus reduced activity in older erythrocytes) in all red cells in hemizygous males and homozygous females. There is variable phenotypic expression in heterozygous females depending on X-inactivation patterns [2]. As with other sex-linked disorders, phenotypic screening of male subjects is the preferred approach to assess G6PD deficiency prevalence and allelic frequency in the population.

The physico-chemical properties of the G6PD protein can be characterized by different techniques (typically electrophoretic mobility and K_m_) and by its enzymatic capacity to reduce nicotinamide adenine dinucleotide phosphate (NADP) to NADPH in the presence of substrates (usually called enzymatic activity). The spectrophotometric assay [3] is considered the “gold standard” for quantitative assessment of enzymatic activity in red blood cells. Simple qualitative tests, such as the Fluorescent Spot Test (FST) [4], are used for population screening but they usually require some basic equipment, electricity and a functioning cold chain for storage of reagents. Point-of-Care (POC) tests have been used in validation studies [5] but have not shown a high sensitivity for the detection of deficient subjects.

In Southeast Asia, the prevalence of G6PD deficiency varies widely among ethnic groups. In Cambodia and Laos the Viangchan variant is the most common among the non-Chinese populations. Prevalence in Laos is approximately 7% and in Cambodia ranges between 1% and 20% depending on ethnicity [6] [7]. Similarly, in Vietnam, G6PD deficiency prevalence varies widely between ethnic groups from less than 3% in the Kinh [8] to 11% in the S'Tieng [9]. In Thailand, the prevalence of G6PD mutations is as high as 20%, the Viangchan variant predominates in the East and the Mahidol variant predominates in the West [10] [11]. In studies performed in Myanmar, the Mahidol variant is the most common variant (>90% of the affected population) with a prevalence between 4% and 12% in the general Burman population [12]. In two ethnic groups from Myanmar that migrated to southern Thailand, G6PD deficiency is caused mainly by the Mahidol variant and no Viangchan variant was detected [13] [14].

Various ethnic groups live in northwestern Thailand along the Myanmar border where malaria is endemic, and although G6PD deficiency is common, no phenotypic or molecular characterization of G6PD variants affecting this population has been performed. In a geographic area where there will be increasing use of primaquine as a gametocytocide and wide scale primaquine deployment for *P. vivax* malaria elimination, we have carried out a population survey in order to estimate the frequency and distribution of G6PD deficiency among 504 unrelated male subjects of mainly Karen and Burman ethnicity

## Methods

### Study area

Outpatient care is provided by the Shoklo Malaria Research Unit (SMRU) in five clinics extending over 200 km of the Thailand-Myanmar border. The catchment area lies predominantly along the Moei River extending as far as 30-40 km inside Myanmar. The population living in border villages is estimated to be over 250,000 [15], consisting mainly of the Karen and Burman ethnicities. Other ethnic groups include the Mon and Yakine. The population is highly mobile and migration patterns influence the ethnic composition of the population attending the different clinics.

### Study population

From September 2011 until November 2011 all males >7 years old presenting to the outpatient clinics were screened for G6PD deficiency. Those with blood transfusions in the last 3 months or who had a blood related brother already in the study were excluded. Screening was performed regardless of diagnosis (unless moderately to severely ill) and all those whom agreed to comply with the study procedures were approached for informed consent. A total of 514 males equally distributed across the 5 clinic sites were screened and included in the study. The subject's ethnicity and his mother's ethnicity were recorded together with the demographic (current address, place of birth, duration of time living along the border, occupation, location of occupation) and past medical history (history of anaemia or blood transfusion and the last malaria episode).

### Sampling and laboratory tests

Capillary blood from a finger prick was obtained and used for malaria testing, G6PD fluorescent spot test (FST, [4]), and human genotyping. Rapid diagnostic tests (RDT) were used for malaria diagnosis and positive tests were analyzed by malaria smears in order to quantify parasitaemia. The FST (R&D Diagnostic, Greece) was performed on site and centrally in the haematology laboratory of SMRU for quality control. DNA extraction was performed using standard column kits (Favorgen Biothec Corp., Taiwan). Genotyping for the Mahidol variant (487G>A) was performed using primers described by [16]. Amplified fragments were analyzed by 1.5% agarose gel; restriction digestion was performed using HindIII enzyme and checked on 3% Nusieve-Agarose gel. Eight samples from phenotypically deficient subjects without Mahidol variant were analyzed first for four common Asian mutations, namely Viangchan (871G>A), Kaiping (1388G>A), Union (1360C>T) and Canton (1376G>T) using the protocol from [10] and then sequenced. Sequencing of exon 2 to exon 13 of the G6PD gene was performed using a modified protocol as described by [5] and results were compared to human G6PD nucleotide sequences from *e!Ensemble* database ref. ENSG00000160211 (transcript G6PD-006). In 24 G6PD deficient subjects quantitative assessment of G6PD activity was performed in triplicate on an additional sample of venous blood depleted of WBC using a CF11 column [17] using a protocol modified from [3] without correction for 6-PGD activity. Haemolysates were obtained by a simple freezing-thaw step overnight at −80°C and analysis performed at 30°C within 30 minutes after thawing; haemoglobin concentrations of samples were assessed using Drabkin's reagent on the same haemolysates. Activity at 37°C was calculated using the conversion factor of the original protocol. Spectrophotometric assay had been already validated in the laboratory before, with a within-laboratory precision of 0.695IU/gHb, assessed following the protocol in [18]. At the condition used, the photometric accuracy of instrument was ±0.002 Abs and photometric range -4≈4Abs (SHIMADZU CORPORATION, Japan). G6PD activity in the normal population of males was assessed before using blood from 34 males analyzed in triplicate (CV<5%) with a median (IQR) of 11.5 (3.3) IU/gHb.

Statistical analysis was performed using STATA SE 11.2 and SPSS v20. The Chi-square test was used for comparisons of allelic frequencies among ethnic groups and parasite prevalence, and Student's t-test was used to compare parasite densities. As the X-chromosome in males is inherited only from maternal side, we have classified subjects based on the reported ethnicity of the mother for the analysis of ethnicity.

Written informed consent/assent was obtained from all subjects prior to study procedures. Written informed consent was obtained from the guardians, next of kin, or caretakers when minors <18 years old were enrolled into the study. Ethical approval was given by the Ethics Committee of the Faculty of Tropical Medicine, Mahidol University (TMEC 11-004) and University of Oxford (OXTREC 18-11).

## Results

A total of 514 unrelated males who attended the five SMRU clinics along the Thai-Myanmar border were included in the study between September and November 2011. The mean (SD) age was 27.0 (13.0) years and the main ethnic groups were Karen (69.8%), Burman (24.5%), Mon (0.6%), and Yakine (1.4%). A small proportion had mixed ethnicity (3.7%). Five samples were rejected due to poor quality and five samples were not transferred from the clinic to the central haematology laboratory giving a final sample size of 504.

### G6PD and malaria

Of the tested subjects, 114 (23%) had malaria, *P. falciparum* in 44 cases (39%) and *P. vivax* in the remaining 70 cases (61%). These proportions were 47% and 53% (8 and 9) in G6PD deficient and 37% and 63% (36 and 61) in G6PD normal subjects respectively (P = 0.44). There was no significant difference in the malaria positivity rate when comparing G6PD normal (97/435, 22%) and deficient subjects (17/69, 25%). Parasite densities were lower in G6PD deficient compared to normal subjects for *P. falciparum* (622/µL and 2210/µL respectively) and *P. vivax* (181/µL and 6331/µL respectively) but these differences were not significant.

### Prevalence and distribution of G6PD deficiency

**Figure 1:**
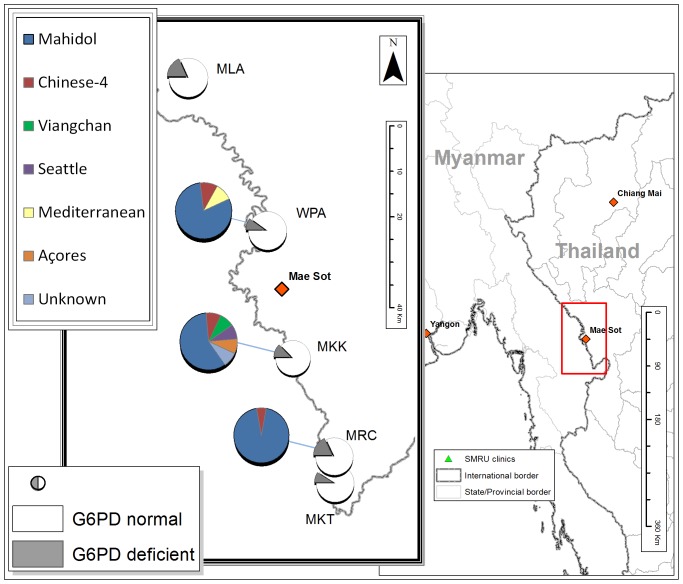
Geographic distribution of G6PD deficient variants detected in five SMRU clinics along the Thailand-Myanmar border. The map indicates the geographic location of the five SMRU clinics where G6PD phenotypes and genotypes were investigated. The white and grey pie charts indicate the proportion of samples taken in each clinic that were either G6PD normal (white) or G6PD deficient (grey). The second-layer pie charts indicate the genetic variants causing phenotypic deficiency at the corresponding site. In two clinics, MLA and MKT, only Mahidol variant was found.

The overall G6PD deficiency frequency by the FST (Table 1) was 13.7% (69/504); 14.1% (50/354) in Karen and 12.9% (16/124) in Burman subjects. The prevalence of G6PD deficiency varied amongst clinic sites from 9% to 18% (Fig. 1), mirroring in part the ethnic composition of the population attending each site.

**Table 1:** Number of subjects according to clinic site, G6PD phenotype and ethnic group.

Clinic site	G6PD	Karen	Burman	Other	Total
Wangpha (WPA)	Total	56	40	4	100
	Deficient	7.1% (4)	15.0% (6)	0.0% (0)	10.0% (10)
Maela (MLA)	Total	106	2	1	109
	Deficient	18.9% (20)	0.0% (0)	0.0% (0)	18.3% (20)
Mawkerthai (MKT)	Total	63	29	10	100
	Deficient	7.9% (5)	13.8% (4)	0.0% (0)	9.0% (9)
Mae Khon Kaen (MKK)	Total	30	52	13	95
	Deficient	10.0% (3)	11.5% (6)	23.1% (3)	12.6% (12)
Moruchai (MRC)	Total	99	1		100
	Deficient	18.1% (18)	0.0% (0)		18.0% (18)
All sites	Total	354	124	28	504
	Deficient	14.1% (50)	12.9% (16)	10.7% (3)	13.7% (69)

The first column indicates the SMRU clinic site where samples were collected. For each ethnic group the total number of subjects included and the relative proportion of G6PD deficient are shown.

### Description of variants

All the 435 phenotypically normal subjects were analysed for Mahidol variant (487G>A) and were normal (wild type genotype) indicating a negative predictive value for the FST of 100% for this variant. Among the 69 subjects with G6PD deficiency by FST, 61 had a confirmed Mahidol variant genotype. Among the remaining 8 subjects analysed by gene sequencing, three had Chinese-4 variant (392G>T), one Viangchan (871G>A), one Açores (595A>G), one Seattle (844G>C) and one Mediterranean (563C>T) (Fig. 1). For one phenotypically deficient sample no mutation was found after sequencing of the coding region; the FST could not be repeated so we consider this either a laboratory error or a deficiency caused by a polymorphism in the promoter or at a splicing site [19]. Among the two major ethnic groups, Burmans showed greater diversity of G6PD deficiency mutations compared to Karen; among the 16 deficient subjects of Burman origin 10 had the Mahidol variant while among the 50 G6PD deficient subjects of Karen ancestry, 49 had Mahidol variant (P<0.01). Five of the 8 non-Mahidol G6PD deficient subjects came from the same clinic (Mae Khon Kaen) accounting for almost half of all the deficient subjects found at this site (5/12). The same site showed the highest variability in terms of ethnic composition with 10% of subjects of mixed ethnicity. Three out of the six G6PD deficient variants found in subjects of Burman ethnicity in this population (Açores, Seattle and Mediterranean), have never been described in the area before.

### Quantitative assessment of G6PD activity

Quantification of G6PD activity by spectrophotometry was performed on a subset of the deficient subjects including 21 samples with Mahidol variant, one with Viangchan, one with Seattle and one with Chinese-4 mutations (Table 2). The activity in the 21 Mahidol samples had a normal distribution with a mean (SD) of 0.059 (0.031) IU/gHb. When compared to the Mahidol variant, Viangchan showed an activity in the same range while Chinese-4 and Seattle variants showed activity almost 10 times higher; although the quantitative assay was performed only in single subjects for the non-Mahidol mutations, all these mutations had a residual activity lower than 10% and they all were correctly diagnosed by the fluorescent spot test.

**Table 2:** Enzymatic activity assessed by the spectrophotometric assay in G6PD normal males and hemizygous males for 4 different G6PD variants.

G6PD genotype	N	Median activity (IU/gHb)	Mean activity (IU/gHb)	SD	95% CI for mean
Wild type	34	11.540	12.114	2.178	11.350–12.880
Mahidol	21	0.067	0.059	0.030	0.045–0.073
Viangchan	1		0.010		
Seattle	1		0.912		
Chinese-4	1		0.839		

The activity in G6PD wild type males was established before in the laboratory. The enzymatic activity was obtained by analyzing each blood sample in triplicate. N indicates the number of subjects who provided the blood for analysis.

## Discussion

G6PD deficiency is common in this malaria endemic region along the northwestern border of Thailand. The Mahidol variant (Gly163Ser) which is the most common G6PD mutation in Myanmar [12], [13] and western Thailand [10] accounted for the majority (88%) of G6PD deficiency. G6PD Mahidol was discovered in 1972 [20] and a molecular characterization was reported in 1989 [21]. Recently a lower capacity of protein folding has been suggested as the cause of enzymatic deficiency [22]. The present study shows that in the population living along the Thailand-Myanmar border, G6PD Mahidol enzymatic activity in hemizygous males is below 10% of the activity detected in G6PD normal males of the same population. Although the quantification was performed in one third of the deficient subjects, these data seem to contradict the historical classification of G6PD Mahidol as a WHO Class III or “mild deficient variant”. The inclusion in the Class III came probably from data reported in the paper by Panich and colleagues [20] where a mutation with residual activity ranging from 5 to 32% (mean 11%) was found in 22 Thai male subjects. The mutation was called “Mahidol” but no analysis of DNA was performed at that time and therefore it is impossible to say whether the subjects analyzed were all carriers of the molecular Mahidol variant or possibly Viangchan, the most common mutation among Thais [10]. In the present study, the phenotypic analyses have been performed using the WHO recommended protocol on samples with PCR confirmed Mahidol mutation; our estimation of residual enzymatic activity is consistent with other published data on samples characterized at the DNA level [13] and it is well below the 10% of normal. This illustrates the limitation of a classification system often based on single observation and non-standardized laboratory methodology and considered “no longer useful” for discrimination of Class II and Class III variants [23].

Chinese-4 variant (Gly131Val, also called Quin Yuan) found in the present study at three different sites and in subjects of different ethnic groups (Karen, Burman and Yakine) has been described before in China and in the Thai population residing in the western regions of Thailand [14] at a population frequency of approximately 1%. Viangchan variant, the most common G6PD mutation in Laos, Vietnam, and Cambodia was found here in a subject of Burman ethnicity. In the present study the analysis of the phenotypically deficient subjects has led to the detection of other 3 uncommon mutations, some of which have never been described in the area. Açores variant (Ile199Val) has been described only in Portugal so far [24]. Seattle variant (Asp282His, also called Lodi/Modena, FerraraII, Seattle-like) has been found repeatedly and characterized in Italy [25] and found in the Canary Islands [26] and in Greece [27]. The Mediterranean variant has been found here and in many distant populations ([28], [29], [30], [31]), and it is a striking example of convergent evolution resulting from malaria selective pressure [32].

Not surprisingly, the molecular heterogeneity of the G6PD gene found in this area is comparable to most of other places where this has been investigated (Cambodia, [5]; West Africa, [33] and [34]; China, [35]). Now that genotyping is relatively easy, G6PD population surveys are often undertaken without phenotypic testing and a panel of mutations are screened; this tends to underestimate the overall prevalence of deficiency in the population level by missing the less common variants; in trials where whole gene sequencing has been performed, even without phenotypic tests, additional heterogeneity has been found at the gene level [36].

In conclusion, the population from Myanmar living in the study area has a high frequency of G6PD deficiency ranging from 9% to 18%. Several G6PD variants were found among subjects of Burman ethnic origin while the Karen ethnic group seems to have a more homogeneous genetic background. The fluorescent spot test correctly diagnosed all the hemizygous males in the study subjects, confirming its reliability in this setting. Quantitative characterization of enzymatic activity in deficient subjects with Mahidol, Viangchan, Seattle and Chinese-4 variants showed a residual enzymatic activity <10% for all the variants. This level of deficiency would be expected to be associated with clinically significant haemolysis during treatment with primaquine with the standard 14 days 30 mg base/day adult dose regimen for radical cure of *Plasmodium vivax*.
